# Controllable Release of Povidone-Iodine from Networked Pectin@Carboxymethyl Pullulan Hydrogel

**DOI:** 10.3390/polym13183118

**Published:** 2021-09-15

**Authors:** Hossam E. Emam, Amina L. Mohamed

**Affiliations:** Department of Pretreatment and Finishing of Cellulosic Based Textiles, Textile Industries Research Division, National Research Centre, Giza 12622, Egypt; alo.mohamed12@hotmail.com

**Keywords:** pullulan, hydrogel, povidone-iodine, control release, kinetics

## Abstract

Povidone-iodine (PI) is a common antiseptic reagent which is used for skin infections and wound healing. The control release of PI is quite important to heal the deep and intense wounds. Herein, the preparation of biodegradable pectin@carboxymethyl pullulan (Pe@CMP) hydrogel was carried out and applied for controllable release of PI. CMP was synthesized by interaction of monochloroacetic acid with pullulan at different ratios. The Pe@CMP hydrogel was then prepared by crosslinking of pectin with CMP in presence of glutaraldehyde as cross linker. After carboxymethylation, COOH contents were enlarged to be 24.2–51.2 mmol/kg and degree of substitution was 0.44–0.93. The rheological properties of Pe@CMP hydrogel were enlarged by increment of pectin ratio. Swelling ratio in water (16.0–18.0%) was higher than that of artificial sweat (11.7–13.2%). Pe@CMP hydrogel containing 20% pectin, exhibited the lowest release and 57.7% from PI was released within 360 min. The biological activity of the released PI was monitored to be highly efficient. The kinetic of release was fitted well to the first ordered reaction and Higuchi models. The mechanism of release was explained by the swelling of hydrogel. The networked structure of hydrogel was opened by swelling and PI was released from the outer pores followed by inner pores, achieving the controllable release.

## 1. Introduction

Hydrogels are porous hydrophilic crosslinking networks in three dimensions shapes. They consist of biopolymers or polyelectrolytes and are swollen in the presence of a lot of water or organic fluids [1,2]. Either physical (hydrogen bonds, chains, and ionic gelation) or chemical methods (covalent bonds) may produce hydrogen [3,4,5]. The porous network may be made of either soft polymers or semi-polymeric structures, with air-drying, cryo-desiccation, and other processes. Macroscopic forms of films, beads, sheets, microspheres and even aerogels are transmitted. To date, many natural promising polymer-based hydrogels have been developed with specific functional interfaces [6].

Hydrogel-based wound dressings can meet essential dressing criteria such as moistening a wound and keeping it moist. Although efficiently absorbing substantial exudate, ensuring adhesive-free coverage of sensitive underlying tissue, and reducing discomfort by cooling with excellent compliance, the atmosphere is preserved [7,8]. More specifically, a hydrogel-based dressing with polymeric networks similar to the extracellular matrix (ECM) can actively intervene in the healing process (e.g., antibacterial agents, growth factors). As a result, hydrogels can be used as an effective delivery vehicle for bioactive agents with high drug-loading capability, allowing for localized and sustained release [7,9]. Natural-based hydrogels made of natural polymers such like sodium alginate, carboxymethyl cellulose, chitosan, chitin, gelatin, and hyaluronic acid are particularly promising biomaterials for wound dressing due to their excellent biocompatibility and biodegradability. Natural polymers special properties, such as hemostasis, anti-inflammation and cell proliferation, contribute to the appeal of wound dressings. These features can help speed up the healing of wounds [7,10].

Pullulan is a water soluble, neutral, and linear polysaccharide and is commonly produced by fermentation broth of fungus *Aureobasidium pullulans* [11]. It is composed from maltotriose units (three glucose units linked with α-1, 4 glycosidic bonds) connected by α-1,6 glycosidic units. Due to its unique linkage pattern, pullulan demonstrates characteristic physical properties such like ability of adhesion, fiber formation capacity and impermeable/transparent biodegradable films [11,12]. Consequently, pullulan is used in diverse applications presented in flocculation, cosmetic and food additives, and blood plasma substitutes [13,14]. Nowadays, pullulan is widely applied in human health purposes [13].

Chemical modification is quite interesting to acquire a specific and unique properties for the polymers. However, few reports were focused on the chemical modification of pullulan and more studies in such topic are still required to insert new advanced functions. Bio-based nano-fibers and food packaging esterified pullulan were synthesized by esterification with different carboxylic acids and octenyl succinic anhydride [15,16,17]. Pullulan was recently cross-linked with poly (vinyl alcohol) and chitosan and for application in self-healing and food packaging, respectively [18,19]. The Carvalho researching group prepared amphiphilic composites-based pullulan by crosslinking pullulan with poly(3-hydroxybutyrate-co-3-hydroxyvalerate) and poly(ε-caprolactone) using click chemistry [20,21]. 

During the dermatological surgeries, as the lesions are usually contaminated with microbes, local application of antiseptic reagents which are effective towards various germs is favored. Antiseptic reagents are much suitable alternative to antibiotics due to their broader antimicrobial spectra than antibiotics [22]. Polyvinylpyrrolidone (povidone)-bound iodine (PI) is a complex of iodine and povidone and acts as a quite active antiseptic reagent [23,24,25]. PI is an active agent against several pathogenic strains including gram-negative and gram-positive bacteria, fungi, protozoa, and several viruses such as H1N1 influenza virus [23,26]. PI (10%) is considered as a first antiseptic for the treatment of the surface skin infections and promoting the wound healing [26,27,28]. On the other hand, the deep and intense wounds take a long time to heal and the burns that have irregular shapes are difficult to be cured. There is still no clinically appropriate remedy for pain relief during wound dressing and the marks left on the skin after wound healing are still undesirable. In response to this issue, Zhang et al. created self-healing hydrogel nanocomposites made of the wound sites, then quickly transferred into an integrated hydrogel that filled the wound area and separated it from the outside world and eventually painless removing the wound site by dissolving it in amino acid solution [6,29].

Hydrogels based on biopolymers are ascribed to be beneficial for drug releasing systems as this type of gels does not require any of chemical reactions which might be deleterious for drug releasing. According to literature, polysaccharides-based hydrogels (pullulan@dopamine, curdluan@polydopamine and dextran@polydopamine) could be prepared to be successfully applied in control release of different drugs [30,31,32]. However, few studies were interested in synthesis of hydrogels based on polysaccharides for controllable releasing of PI. It was reported that bio-polymeric dressing based on sodium and potassium salts of alginate could be successfully exploited for controlled release of PI [33]. Another study was considered with the preparation of hydrogels of carboxymethyl cellulose (CMC)/poly (vinyl alcohol) (PVA)/gelatin and cross-linked polyacrylamide (PAM) to be applicable for wound dressing with controllable releasing of PI [34]. In another approach [35], bio-elastomers based starch was also functionalized for PI releasing. Fabrication of chitosan with Polyvinylpyrrolidone for preparation of dressing with regulated release of PI was also studied [36]. Moreover, wound dressing made of bacterial cellulose was most recently applied for controllable release of PI [37]. 

Owing to the essential requirement for the issue of controllable releasing for antiseptic reagents in the wound-healing process, in the current study, synthesis of biodegradable hydrogel-based pullulan and pectin was uniquely investigated for controllable releasing of PI. Carboxymethyl pullulan was firstly synthesized and cross-linked with pectin by crosslinking agent to obtain pectin@carboxymethyl pullulan (Pe@CMP) hydrogel. The prepared CMP and Pe@CMP hydrogels were investigated by infrared, scanning microscope and energy dispersive X-ray. Rheological and swelling properties are both measured for the prepared materials. The release behavior of Povidone-iodine (PI) from the synthesized Pe@CMP hydrogels were studied in the artificial sweat. Kinetics of the release were measured by different models and the releasing mechanism was suggested and presented. 

## 2. Materials and Methods

### 2.1. Experimental Session

#### 2.1.1. Materials

Pullulan (MW = 200.00) was provided from natural Lab (USA). Pectin (from citrus fruits, degree of esterification = 30, MW = 194.14), Monochloroacetic acid (MCA), Povidone-iodine (PI, 10%) and Glycerin were purchased from Sigma-Aldrich. Sodium hydroxide, Glutaraldehyde (25% aqueous solution, GA) and Isopropyl alcohol (90%) were supported from Fluka. All chemicals were used as received without any purification.

#### 2.1.2. Methods

##### Preparation of Carboxymethyl Pullulan

Carboxymethylation reaction of pullulan was carried out in an aqueous–organic media at alkaline pH. Briefly, in a three-necked round flask, equipped with a mechanical stirrer and condenser, 20 g of pullulan was suspended in 50 mL isopropanol under constant stirring for 30 min at room temperature. Afterward, 40 mL of an aqueous solution of NaOH (1 M) was added drop wisely in 20 min and followed by stirring for an additional 60 min. Different amounts of monochloroacetic acid (20, 30 and 40 g) were drop wisely added to the mixture in 15 min and the reaction was proceeded at 70 °C for 5 h under continuous stirring. The produced carboxymethyl pullulan (CMP) was collected by filtration, then dialyzed against distilled water until neutralization. Finally, the produced CMP was lyophilized and dried under pressure using vacuum oven. The samples ascribed as CMP1, CMP2 and CMP3 according to the amount of monochloroacetic acid added. 

##### Preparation of Pe@CMP Hydrogel

To produce hydrogels, the produced CMP was reacted with pectin (different ratios) in presence of glutaraldehyde (GA) as a crosslinking agent. Briefly, at the beginning, a solution of 5% (*w*/*w*) of each polymer (CMP and pectin) was prepared under continuous stirring at room temperature. After complete dissolving, different ratios between carboxymethyl pullulan and pectin have been mixed under constant stirring, then the reaction was proceeded at 50 °C. After 60 min, 5 mL glycerin (10%, *w*/*w*) and 10 mL of GA (25% aqueous solution) was gradually added with continuous stirring followed by addition of 2 mL HCl to provide the acidic condition for crosslinking the polymers. The mixtures were vigorously stirred, and the reaction was continued at 50 °C for 24 h. At the end, 30 mL of methanol was added to the mixtures and then washed several times with distilled water to remove the unreacted materials. The precipitates were then filtered and dried under freeze drying to produce Pe@CMP hydrogels. The produced hydrogels were labelled as Pe@CMP1, Pe@CMP2 and Pe@CMP3 hydrogels according to the ratio between pectin and CMP (10:90, 20:80 and 30:70). For comparison, two reference gel samples (pectin alone & CMP alone) were separately prepared by the same process to obtain pectin gel and CMP gel.

### 2.2. Bioactivity of PI 

The bioactivity of the PI after releasing was examined through testing the antimicrobial activities of the released PI against two different targeted pathogens using well diffusion test method [38]. The selected pathogens were *Escherichia coli* ATCC-25922 (*E. coli*, gram—ve bacteria) and *Candida albicans* ATCC-10231 (*C. albicans*, fungi). The microbial pathogens were kept at 4 °C in the nutrient agar and then part from agar was scraped in the middle of plate. A total of 100 µL from the released PI was dropped in the scratched area. The plates were incubated at 37 °C overnight and the inhibition zone diameters around the scratched area were recorded in mm.

### 2.3. Characterization and Analysis

#### 2.3.1. Carboxylic Content and Degree of Substitution 

The content of carboxylic (COOH) group is an important factor indicated for the carboxymethylation process. The COOH contents for the synthesized CMP samples were measured by using the methylene blue (C.I.Nr. 52015, Merck) dye method [39,40]. A certain dried weight (0.08–0.1 g) from the samples were moved to a 50 mL round flask. To the samples, 25 mL from both of borate buffer solution (pH = 8.5) and methylene blue (300 mg/L) were added and then shaken at room temperature for 24 h. 2.5 mL was withdrawn from the mixture and transferred to 50 mL measuring flask followed by addition of 5 mL HCl solution (0.1 M) and then completed the total volume by distilled water. The absorbance of the solution was measured by using a spectrophotometer (Cary 100 UV-Vis-NIR Systems, from Agilent) at maximum wavelength (λ = 664.5 nm). The COOH contents were calculated according to Equation (1). For each sample, 3 repetitions were carried out and the mean values were reported. The substitution degree (DS) for CMP samples were measured by using the values of COOH contents.

(1) $$C_{\mathrm{COOH}}=\frac{(C_{1}-C_{2})\times 0.00313}{m}$$

where *C*_COOH_ is the carboxylic content (mmol/kg), *C*_1_= methylene blue concentration for the blank without sample (mg/L), *C*_2_ = methylene blue concentration for the samples (mg/L) and *m* = weight of samples (g).

#### 2.3.2. Infrared Spectra (FTIR)

All the synthesized CMP and Pe@CMP hydrogels were investigated by attenuated total reflection-Fourier transform infrared spectroscopy (ATR-FTIR, Jasco FT/IR 6100) connected to a detector of deuterated triglycine sulfate (TGS) and accessories of the attenuated total reflectance (ATR unit with Golden Gate diamond crystal). The absorbance spectra were collected in the wavenumber range of 500–4000 cm^−1^, using resolution of 4 cm^−1^ with 1 cm^−1^ scanning interval and smoothed with 15 points.

#### 2.3.3. Scanning Electron Microscopy (SEM)

The morphological features for the dried samples of CMP and Pe@CMP hydrogels were examined by using a high-resolution scanning electron microscopy (HRSEM, SEM Quanta FEG 250 with field emission gun, FEI Company—The Netherlands). Samples in the powder form were mounted on the stabs and coated with thin layer from gold and then conducted to the microscope. The chemical compositions of the samples were performed by surface energy dispersive X-ray spectroscopy (EDX, EDAX AMETEK analyzer) attached with the microscope. 

#### 2.3.4. Rheological Properties

The rheological properties of the synthesized gels (pectin, CMP, Pe@CMP) were examined at 25 ± 0.2 °C by using a coaxial rotary viscometer (HAAK V20—Germany). According to Newton’s law, the apparent viscosity (η; cP) is defined as the coefficient of shear stress (τ; dyn/cm^2^) divided by the shear rate (γ; s^−1^) [41].

#### 2.3.5. Solution Uptake and Swelling

Solution uptake of the synthesized gels (pectin, CMP, pe@CMP) was measured in water, sodium chloride (0.1 M) and artificial sweat (pH 6.5) solution. The artificial sweat (pH 6.5) was synthesized in accordance with the recipe of EN1811-1999 [42] by using sodium chloride (1.08% (*w*/*v*) and the pH was adjusted to 6.5 using lactic acid and urea. A definite weight of 1 g from the samples was placed in vowel bag and then dropped in a measuring cylinder containing 100 mL water or sodium chloride solution or artificial sweat at 25 °C. At different interval immersion time (1, 6, 12, 18 and 24 h), the vowel bag was picked up from the solution and left for 5 min to drain out the excess of solution. The weight of vowel bag was measured and solution uptake was calculated according to Equation (2). The swelling ratios of the samples were estimated from Equation (3).

(2) $$SU=\frac{\left(W_{t}-W_{b}\right)-W_{p}}{W_{p}}$$

(3) $$S\%=\frac{\left(W_{t}-W_{b}\right)}{W_{p}}\times 100$$

where *SU* is the solution uptake (mg/g) and *S*% is the swelling ratio. *W_b_* and *W_t_* are the weight of the blank vowel bag and vowel bag containing samples after water treatment, respectively. *W_p_* is the weight of the dried gel sample.

### 2.4. Release of Povidone-Iodine

Povidone-iodine (PI) was selected in this study, because it is a common antiseptic agent which normally used for skin disinfection [43]. Firstly, PI was loaded onto the prepared hydrogels (CMP & Pe@CMP) as follows; certain weight of 0.5 g from hydrogel samples was soaked in 2% of PI solution for overnight. The samples were taken out and left for 10 min to leach out the un-bonded PI. The PI-loaded hydrogel samples were then immersed in artificial sweat solution (pH 6.5) at 25 ± 1 °C and the release behavior of PI from hydrogel network was studied. At interval time (0–360 min), 3 mL liquor was withdrawn from the solution and absorbance spectra were measured by UV-Vis spectrophotometer (Cary 100 UV-Vis-NIR Systems, from Agilent). The concentration of PI released from hydrogel to the environment was calculated from the intensity of absorbance and by using the calibration curve. The experiment was repeated twice and the mean values were recorded. The artificial sweat was prepared according to BS EN1811-1999, with pH 6.5. As solution composed from 1.08% sodium chloride (*w*/*v*) and pH was adjusted to 6.5 by urea and lactic acid [42,44].

### 2.5. Statistical Analysis

The swelling, solution uptake and the release of PI experiments presented in the current study were carried out three times and the average values were considered. Kinetics parameters of the release, determination coefficient (R^2^) and error bars, were all calculated by using 2016 Microsoft Excel. 

## 3. Results and Discussion 

### 3.1. Synthesis of Pe@CMP Hydrogel

**Figure 1:**
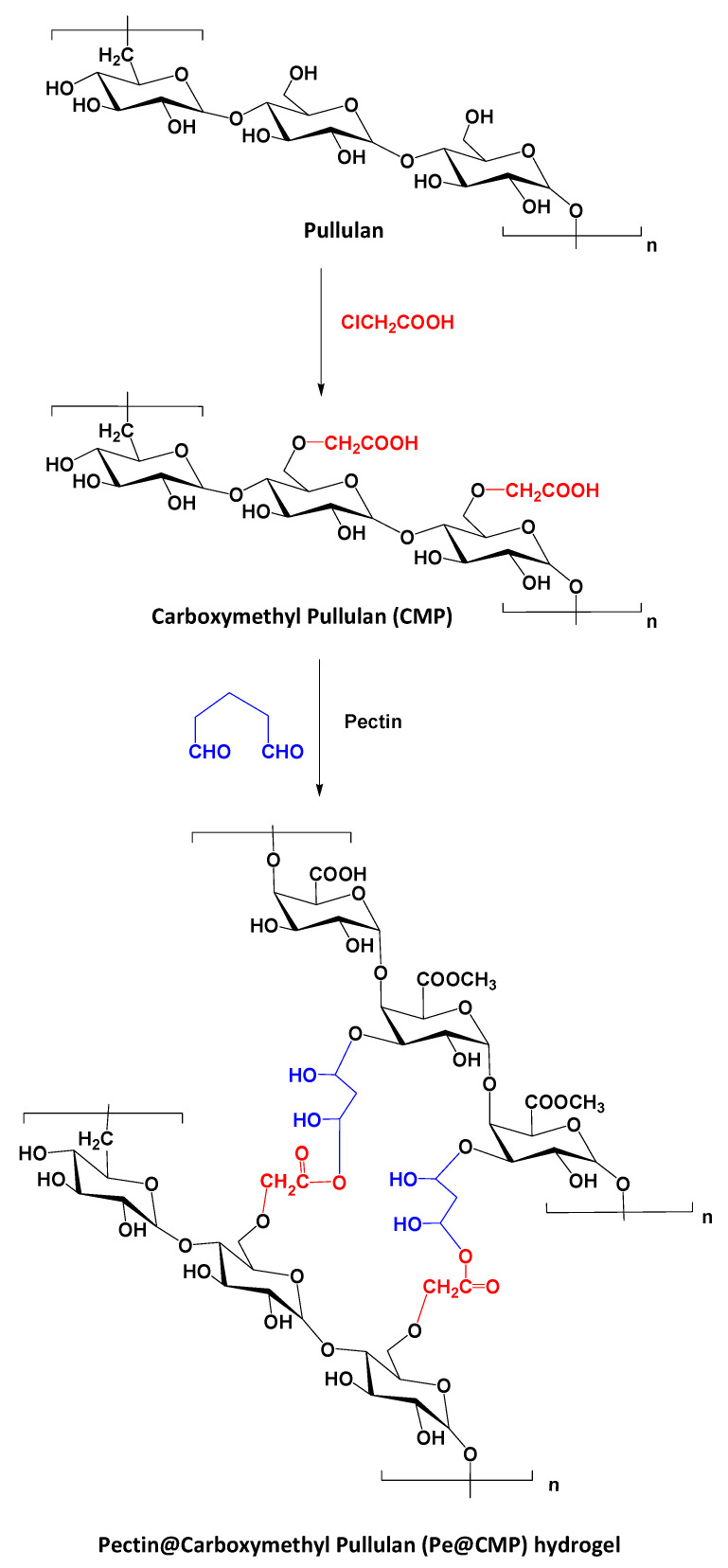
Schematic preparation of Pe@CMP hydrogel.

Carboxymethyl pullulan “CMP” was firstly synthesized by interaction of pullulan and monochloroacetic acid with different ratios through etherification reaction as presented in Figure 1. In principle, monochloroacetic acid reacted with the hydroxyl groups (OH) of pullulan through substitution reaction forming the mono-ether. By changing the ratio between pullulan and monochloroacetic acid, the degree of substitution in CMP changed [45]. The substitution reaction is suggested to take place firstly on the accessible OH- group of C6 as primary alcohol followed by the secondary alcoholic groups of C2 and C3. Due to the carboxymethylation process, by increasing the ratio of monochloroacetic acid, the carboxylic content increased logically for the modified pullulan “CMP” owing to the increment in degree of substitution “DS”. The molar mass of carboxylic content was measured for the series of CMP as summarized in Table 1. COOH content was considerably increased after etherification reaction as a result of the insertion of -CH_2_-COOH groups. The enlargement in COOH content was directly proportional to the content of monochloroacetic acid exploited in preparation of CMP series. COOH contents were increased from 2.2 mmol/kg for pullulan to 24.2–51.2 mmol/kg for CMP. Values of DS were calculated from the estimated COOH contents and they were enlarged with the ratio of monochloroacetic acid until reached 0.88 for CMP3. The further increment in monochloroacetic acid ratio, is logically accompanied with the increment in DS values and consequently the COOH contents. 

The prepared CMP was cross-linked with pectin as neutral polysaccharide in the presence of glutaraldehyde as cross-linking agent to give pectin@carboxymethyl pullulan (Pe@CMP) composite hydrogel. Glutaraldehyde acted as cross-linker, bonded with CMP from a side and with pectin from another forming networked Pe@CMP hydrogel. COOH groups of CMP and OH groups in pectin are both cross-linked via glutaraldehyde molecules (Figure 1). The increment in the DS of CMP improved the crosslinking through the enlargement in the freely accessible COOH groups and, consequently, highly oriented networked Pe@CMP hydrogel generated. Different ratios between pectin (10, 20, 30%) and CMP (90, 80, 70%) were studied to optimize the best hydrogel formulation (Pe@CMP1, Pe@CMP2, Pe@CMP3).

#### 3.1.1. FTIR Spectra

**Figure 2:**
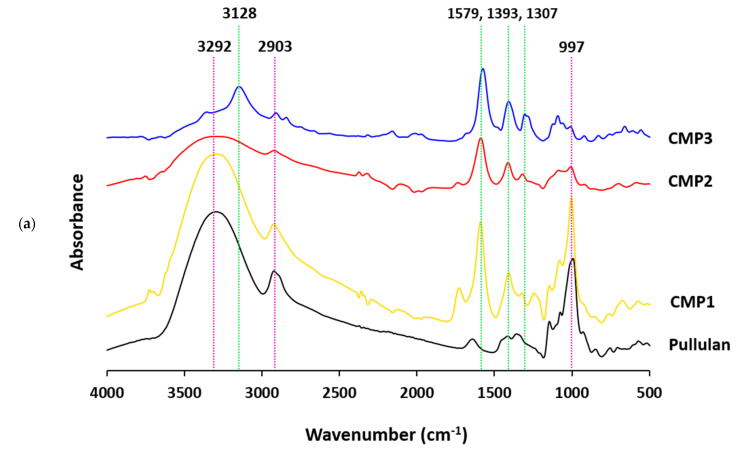

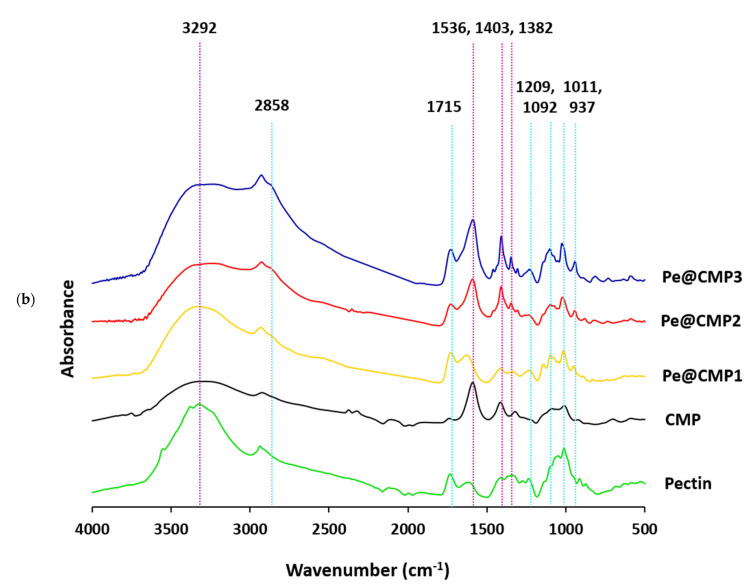
FTIR spectra for the prepared; (**a**) CMP and (**b**) hydrogels.

The infrared spectra were analyzed for pullulan, CMP and Pe@CMP hydrogel in order to follow up the chemical interchanging due to the modification, while IR spectra were displayed in Figure 2. Pullulan exhibited several absorbance peaks at 3292, 2903, 1627, 1393/1307 and 997 cm^−1^ (Figure 2a). These vibrational peaks are referred to the hydroxyl (OH) group, aliphatic asymmetric stretching aliphatic CH_2_, O-C-O stretching, stretching of C-O and bending of OH, respectively [17,18,19]. After carboxymethylation process, new vibrational peak was appeared at 1579 cm^−1^ corresponding to carbonyl (C=O) group. The intensity of C-O stretching (1393/1307 cm^−1^) was significantly increased due to the carboxymethylation, while the vibrational peaks of OH (3292 and 997 cm^−1^) and C-O (1627 cm^−1^) were diminished after etherification due to the contribution in carboxymethylation. At high DS, blue shifting in the vibrational peak of OH was observed. IR spectral observation confirmed the etherification reaction of pullulan and consequently preparation of CMP. 

IR spectra for the Pe@CMP hydrogels were presented in Figure 2b. For pectin macromolecule, the characteristic vibrational peaks were appeared at 3312 cm^−1^ (O-H), 2925 cm^−1^ (CH_2_ aliphatic), 1715 cm^−1^ (C=O), 1595 cm^−1^ (C-O) and 1011 cm^−1^ (O-H bending) [46,47]. For the Pe@CMP hydrogel, the vibrational peaks of pectin and CMP were appeared. Additionally, new vibrational peaks for the glutaraldehyde cross-linker were observed at 2858 cm^−1^ (CH_2_ aliphatic), 1403 (C-O), 1209 cm^−1^ (C-H bending), 1092 cm^−1^ (O-H bending) [48]. By increasing the ratio of pectin in hydrogel, the intensities of vibrational peaks for pectin and glutaraldehyde were increased which reflected the increment in crosslinking reaction and consequently the crosslinking ratio. These vibrational spectral results declared the cross-linking reaction between CMP and pectin through glutaraldehyde for further confirmation of the successive preparation of Pe@CMP hydrogel. 

#### 3.1.2. Micrographs

**Figure 3:**
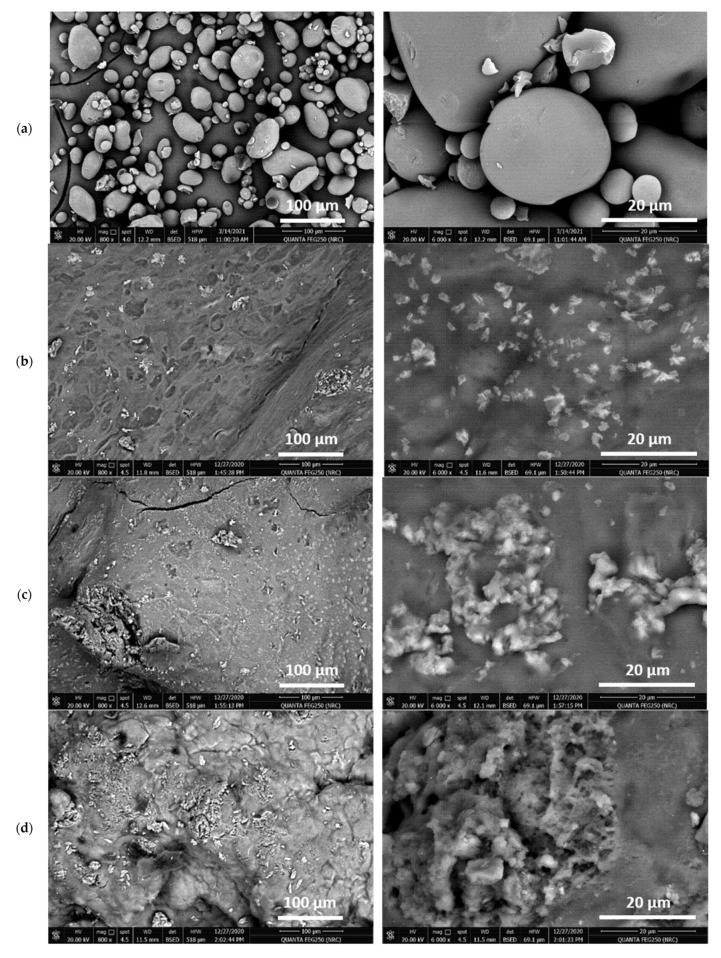
Micrographs for the prepared materials and hydrogel; (**a**) CMP, (**b**) Pe@CMP1, (**c**) Pe@CMP2 and (**d**) Pe@CMP3.

The morphological characters of CMP and Pe@CMP hydrogels at different ratios were showed in Figure 3. The micrographs clarified that; micro-balls structured CMP was observed with large size distribution of 1.6–41.0 µm. The cross-linked Pe@CMP hydrogels were seen with microspores surface filled with pectin particles. The porous surface is related to the networked gel formation of Pe@CMP composite. By increasing the ratio of pectin in the formed hydrogels, the formation of hydrogel clearly appeared, and the pores were observed with sponge-like structure. For all samples (CMP & Pe@CMP hydrogel), the elemental analysis of EDX (Figure S1, Supplementary material) showed signals of carbon and oxygen only which were corresponded to the chemical compositions of the samples. 

#### 3.1.3. Rheological Properties 

**Figure 4:**
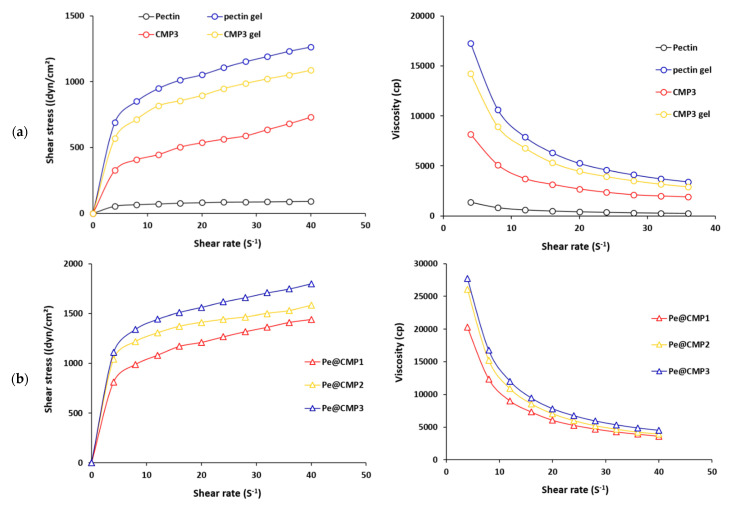
Rheological properties for the pectin, CMP and Pe@CMP hydrogel; (**a**) pectin & CMP and (**b**) Pe@CMP hydrogel.

Rheological properties are characterized for the fluid materials which exhibit the fluid resistance to flow. Hence, the rheological properties are measured for the synthesized CMP and hydrogels. The results of shear stress and viscosity as function of shear rates were figured in Figure 4. For all tested samples, the shear stress increased, and the viscosity decreased with shear rate. For CMP3 and CMP3 gel, shear stress was significantly enlarged from 326 and 570 dyn/cm^3^ to 731 and 1087 dyn/cm^3^ by raising the shear rate from 4 to 40 S^−1^, respectively. Much higher shear stress (1265 dyn/cm^3^) was observed for pectin gel. At 4 S^−1^, the viscosity for pectin, pectin gel, CMP3 and CMP3 gel were 1364, 17,237, 8151 and 14,245 cp, respectively. This means that the formation of gels (pectin or CMP) in presence of glutaraldehyde as cross-linking was accompanied by considerable increment in rheological properties. In case of the synthesized Pe@CMP hydrogels, values of shear stress were much higher than that of pectin and CMP and reached 1443–1801 dyn/cm^3^ at 40 S^−1^. The viscosity of Pe@CMP hydrogels was frequently higher (20,301–27,743 cp). The higher rheological properties of Pe@CMP could explain the formation of hydrogel network and declared the thixotropic fluid character of hydrogels [49,50]. The rheological properties of Pe@CMP hydrogels were insignificantly changed by increment the ratio of pectin and, hence, the ratio of 20% pectin was sufficient to obtain an appropriate Pe@CMP hydrogel. 

#### 3.1.4. Solution Uptake and Swelling Properties 

**Figure 5:**
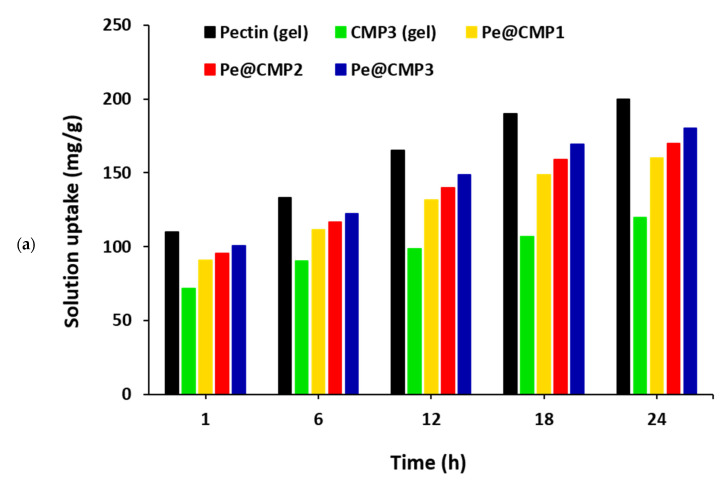

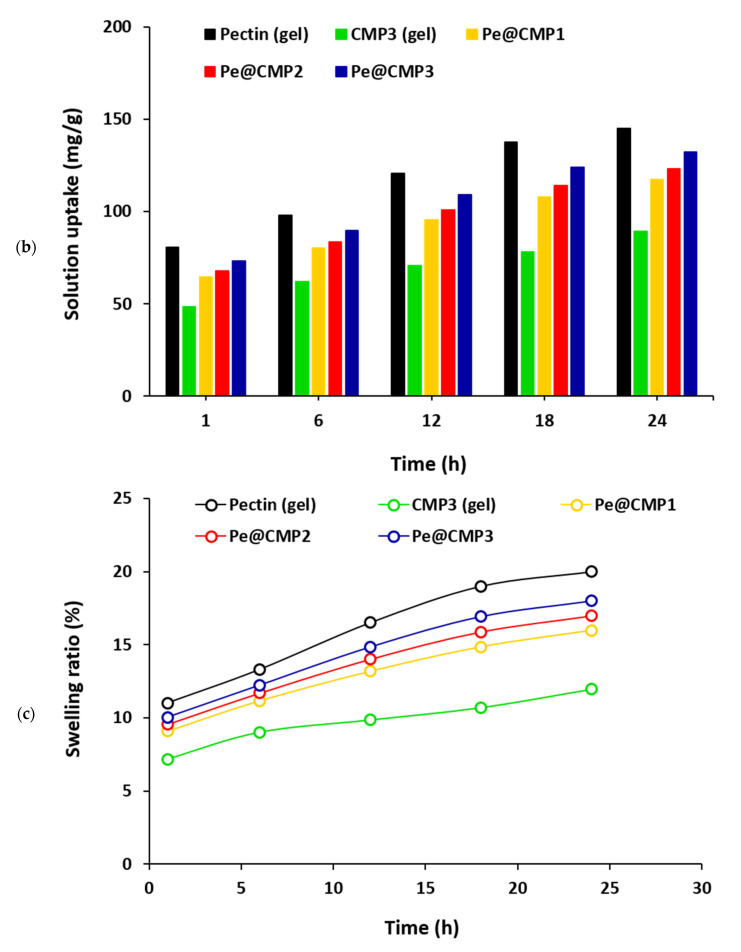

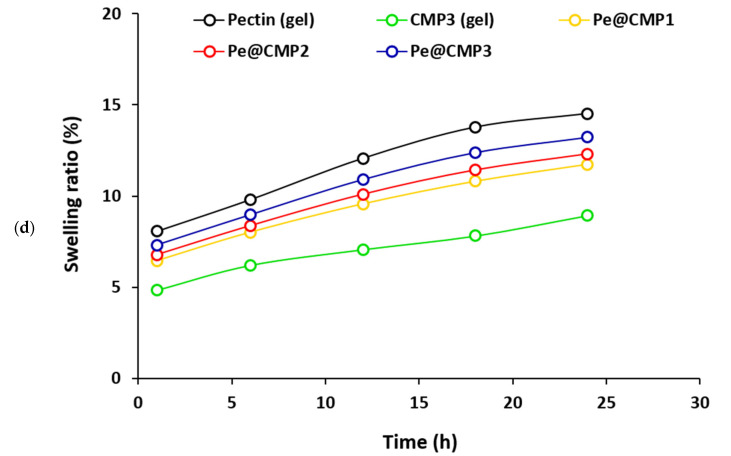
Solution uptake and swelling ratio for the pectin, CMP and Pe@CMP hydrogel; (**a**) water uptake, (**b**) artificial sweat uptake, (**c**) swelling in water and (**d**) swelling in artificial sweat.

The release properties of any materials are functions of swelling behavior which are expressed as solution uptake. Therefore, solution uptake was firstly measured for the all prepared gels (pectin, CMP and Pe@CMP) and the results were presented in Figure 5a,b. The solution uptake was studied for three different medium: water, sodium chloride and artificial sweat. For all tested samples, the water uptake was higher than that of artificial sweat uptake by factor of 1.3–1.5, while the NaCl uptake was quite lower than that of water by 2–6 times (Figure S2, supplementary file). The solution uptake was gradually increased with immersion time. The increment of uptake was quite fast in the first 6 h and it was insignificant after 12 h. The pectin and CMP gels showed the highest and the lowest solution uptake, respectively. The Pe@CMP hydrogel exhibited more suitable solution uptake which is located between that of pectin gel and CMP gel. The increment of pectin ratio in Pe@CMP hydrogel was accompanied with raising the solution uptake. For Pe@CMP2, the water and artificial sweat uptakes were 116.9 mg/g and 83.7 mg/g, respectively, after 6 h of immersion time. 

The swelling properties of the Pe@CMP composite hydrogels were then studied at different time up to 24 h in water, NaCl and artificial sweat medium as represented in Figure 5c,d and supplementary file. The swelling ratio was gradually increased by the immersed time and the difference in the swelling ratio was not significant by increment the pectin ratio in Pe@CMP hydrogel. The swelling in water was raised from 9.6–10.0% to 16.0-18.0% from extended the immersed time from 1 h to 24 h. The swelling of Pe@CMP hydrogels in artificial sweat was rationally lower compared to water and reached only 6.5–7.3% and 11.7–13.2% after 1 h and 24 h, respectively. In comparison with Pe@CMP hydrogels, the CMP gel showed quite low swelling properties, as the swelling ratios were 12% and 8.9% in water and artificial sweat, respectively, within 24 h contact time. The solution uptake and swelling properties were both supported each other and confirmed the formation of cross-linked Pe@CMP hydrogel with networked structure. The cross-linked network skeleton permitted the solution to go into the pores and increased the dimension causing swelling behaviour which is very important character for drug release applications. Compared to artificial sweat, uptake and the swelling in water medium were significantly higher which explained by the formation of hydrogen bonding between water molecules and functional groups (-O- and -OH) of Pe@CMP hydrogel.

#### 3.1.5. Release Properties of Povidone-Iodine (PI) 

**Figure 6:**
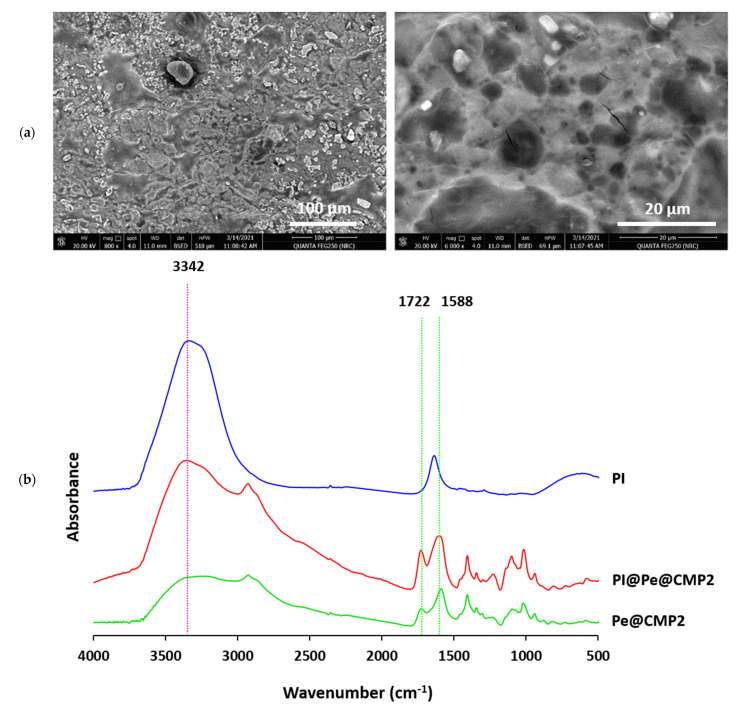
(**a**) Micrographs and (**b**) FTIR for the PI@Pe@CMP.

Povidone-iodine (PI) antiseptic uses for skin disinfection and, hence, controlling in its release are quite important to regulate the maximum benefit. Herein, the releasing of PI was studied in the artificial sweat (pH = 6.5) because PI is used for skin treatment. Firstly, the PI was loaded upon the synthesized hydrogels (CMP and Pe@CMP) and the loading was confirmed by SEM and FTIR spectra as shown in Figure 6. The micrograph for PI@Pe@CMP hydrogel showed that the PI particles were distributed on to the surface of Pe@CMP and filled the pores. The elemental analysis of EDX (Figure S1, supplementary file) recorded the signal of I beside that of O and C for hydrogel. In FTIR spectral analysis, absorption peak of C=O in PI (at 1627 cm^−1^) and was overlapped with that of Pe@CMP hydrogel [51,52]. Both of micrographs and FTIR spectra confirmed the loading of PI onto the hydrogel. 

**Figure 7:**
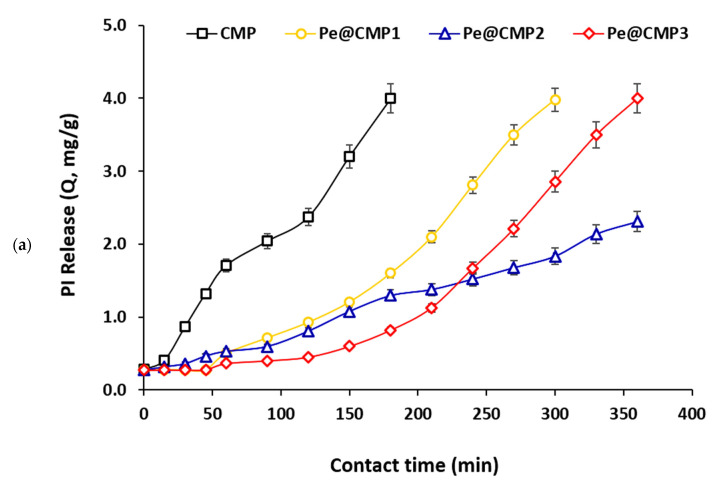

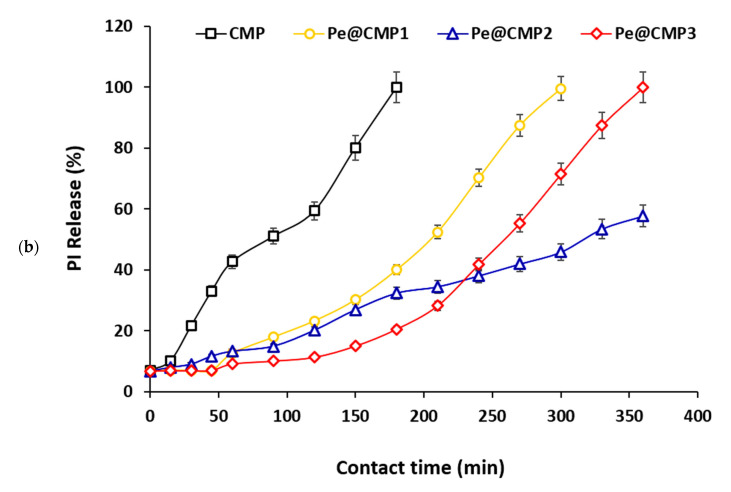
Release behavior of PI in artificial sweat from CMP and Pe@CMP hydrogel; (**a**) release amount and (**b**) release percentage.

The release properties of PI from the hydrogels were studied as presented in Figure 7. The release of PI was estimated by amount (mg/g) and by percentage (%). The release of PI was quite fast in case of CMP gel and PI was completely released within only 180 min. Meanwhile, the Pe@CMP hydrogel showed quite slow release. The release of PI was significantly slowed down by increment the ratio of pectin in hydrogel from 10% to 20% due to the increment in formation of networked structure that could be more beneficial as slow release could give more of opportunities and longer duration for acting as biocidal laborer. Further raising the pectin ratio in Pe@CMP hydrogel to 30% was resulted in faster release and the PI was fully released within 360 min. The higher ratio of pectin in hydrogel was accompanied with increment in the crosslinking with CMP and further helped in network formation which increased the linkages between chains. The further increment in crosslinking was led to decrease the extension of pores and consequently accelerated the release. Hence, the higher networked Pe@CMP hydrogel was not favored and the synthesized hydrogel with 20% pectin (Pe@CMP2) was showed the appropriate releasing behavior. After 360 min, 57.7% of PI was only released from the Pe@CMP2 hydrogel. The rest of PI (42.3%) could be released in another 360 min. These data confirmed the control release of PI from the Pe@CMP2 hydrogel which was carried out in ca. 12 h. 

#### 3.1.6. Kinetic and Releasing Mechanism

**Figure 8:**
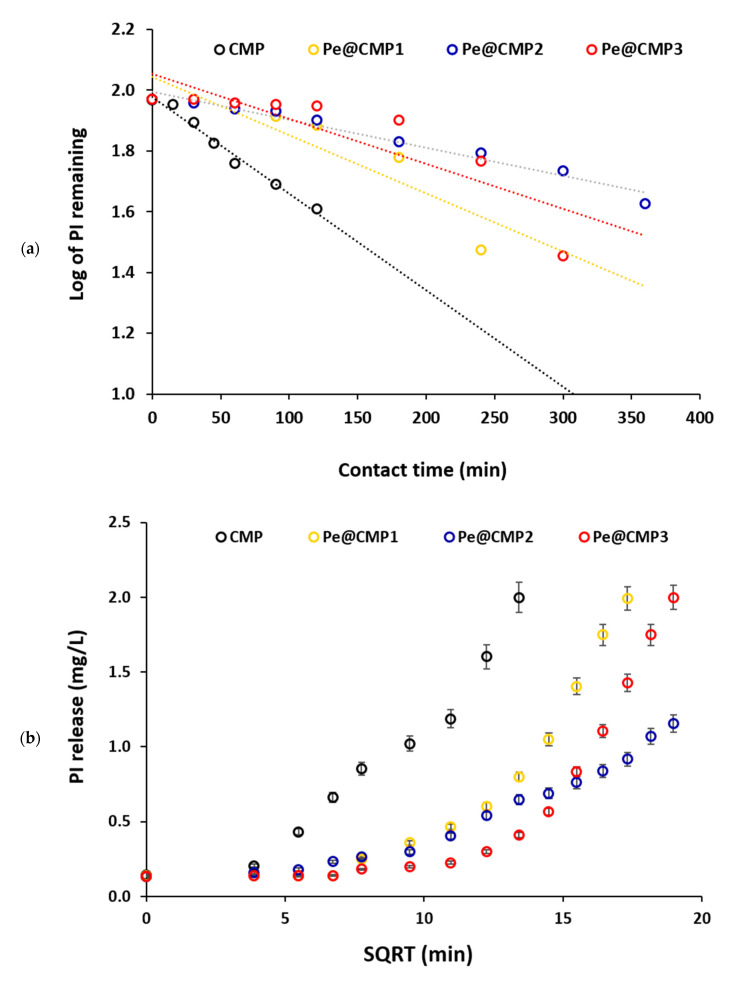

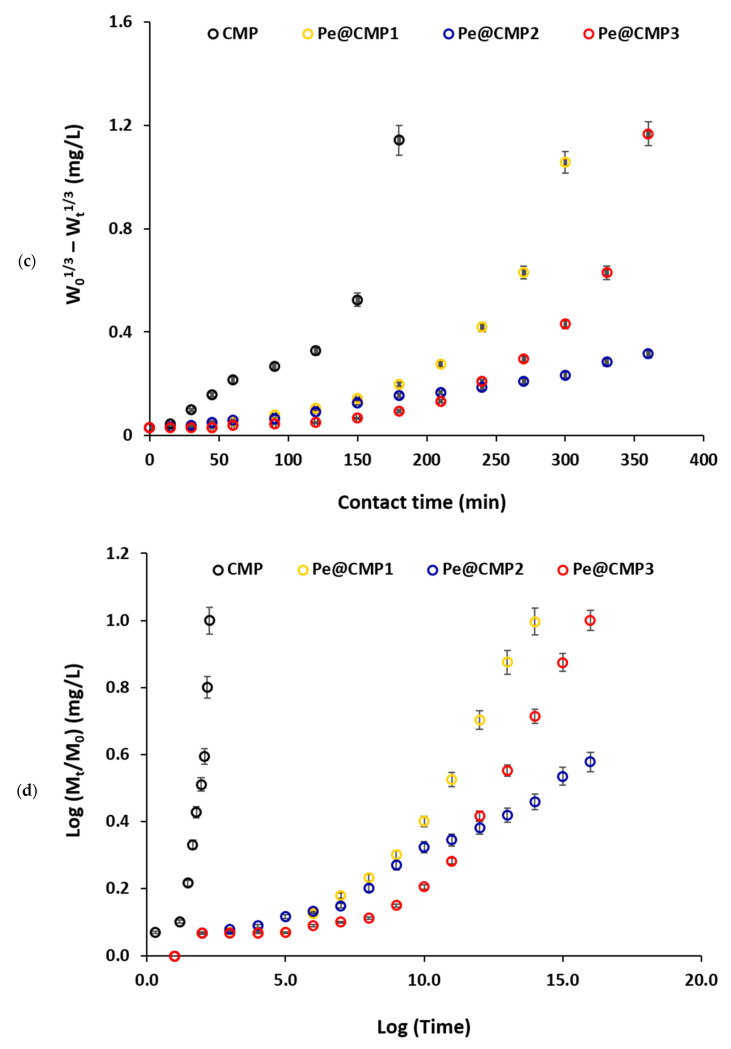
Kinetics for the release of PI from CMP and Pe@CMP hydrogel; (**a**) First, order fitting, (**b**) Higuchi, (**c**) Hixson–Crowell and (**d**) Krosmeyer–Peppas.

The release of PI from the synthesized hydrogels was kinetically studied in order to perform the mechanism of release. In this study, four different kinetic models were applied for the release of PI including first order, Higuchi, Hixson–Crowell and Korsmeyer–Peppas, as shown in Figure 8. For first order kinetic mode, the log of PI remaining percent in the hydrogel was plotted versus the releasing time (Figure 8a). In case of the Higuchi model, the PI release percent was figured out with the square root of releasing time (Figure 8b). The cubic root of the releasing amount for PI was plotted with the releasing time, for the Hixson–Crowell model (Figure 8c). In case of the Korsmeyer–Peppas model, the log of PI release amount is drawn with the log of time (Figure 8d). All of the kinetic parameters for all models (k_1_, k_H_, k_HC_, n, k_kp_ and R^2^) were calculated and collected in supplementary file (Table S1). 

From the data in supplementary file (Table S1), the release of PI from the prepared Pe@CMP hydrogels is followed the first order model and Pe@CMP showed the best fitting owing to obtaining the highest linearity with highest coefficients correlation (R^2^ = 0.99). This fitting indicates that PI release is almost depended on its concentration or its content onto the hydrogel. The constant of releasing rate (k_1_) affirmed that the release of PI from Pe@CMP hydrogels (1.69–0.79 h^−1^) is significantly slower than that from CMP (2.75 h^−1^) and Pe@CMP2 hydrogel showed the slowest rate of PI release (0.79 h^−1^). The release of PI was very close to the iguchi kinetic model with R^2^ ≥ 0.88, which reflects the rational slower rate of diffusion at longer releasing time assigning for the square root of releasing time [53]. Compared to CMP, the rate contestant of Higuchi (K_H_) for Pe@CMP hydrogels are considerably smaller. Pe@CMP2 exhibited the smallest K_H_ which confirmed its slowest diffusion rate at longer time. For the Hixson–Crowell model, weak fitting and weak linearity (R^2^ ≥ 0.75) were observed which reflected that the surface area of the hydrogel is a function of the releasing time [54]. The data fits quite well with the Krosmeyer–Peppas model (R^2^ ≥ 0.87) and the estimated exponential n values were ranged in 0.049–0.077.

Biocompatible hydrogel-based polysaccharides (pullulan@dopamine, curdluan@polydopamine and dextran@polydopamine) were recently synthesized and applied in control release of different drugs [30,31,32]. Meanwhile, few works were studied the release of PI from materials-based biopolymers including alginate, starch and chitosan [33,35,36]. PI was completely released into distilled water from Na-alginate films and Ca-alginate beads within 20 min. and 4 h, respectively [33]. Chitosan based hydrogel showed fast release of PI within 3 h in PBS medium at 37 °C [36]. Meanwhile, quite slower release of PI (7 days) was observed in water from the bio elastomers-based starch [35]. By comparing with the data reported in literature [33,35,36], the applied Pe@CMP hydrogel showed slower PI release than the formerly used Na-alginate film, Ca-alginate beads and bio elastomer-based starch. Additionally, the release of PI from the prepared Pe@CMP hydrogel (57.5%, 6 h) was quite reasonable compared to that of bioelastomer based starch (7 days). 

**Figure 9:**
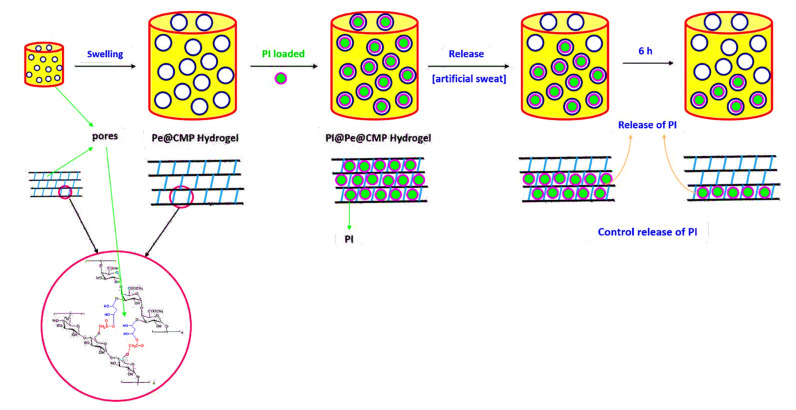
Physical releasing mechanism of PI from Pe@CMP hydrogel.

According to the literature [55], the releasing mechanism of any materials from polymeric matrix could be carried out via chemical or physical way (solute diffusion, degradation of material or swelling of polymer matrix). The PI was loaded onto the Pe@CMP hydrogel by the physical deposition within the pores of network during swelling which suggested the releasing mechanism was more relevant to polymer swelling. The available hydroxyl groups in the Pe@CMP hydrogel might be physically bonded with the PI through hydrogen bonding [56,57]. Thereof, the releasing mechanism of PI from the hydrogel was proposed to be physical process in as suggested in Figure 9. The huge difference in the release of PI between CMP and Pe@CMP hydrogel confirmed that the pores in networked structure was the main affected factor and, consequently, the physical release process was the main releasing mechanism. The higher swelling ratio for Pe@CMP hydrogel confirmed this hypothesis. Moreover, n values in the Krosmeyer–Peppas model (0.049–0.077) notified that the release of PI could not be related to a diffusion/erosion [55] and further supported the swelling mechanism. In conclusion, when the PI@Pe@CMP hydrogel immersed in the artificial sweat, swelled followed by opening the networked structure and the PI was then released from the pores. The PI molecules were released firstly from the outer pores and then from the intercellular pores and, finally, from the bulk pores. Hence, the slow release of PI from hydrogel was obtained achieving the control release process. For Pe@CMP2, 60% from PI was released during 6 h and, subsequently, the full release of *PIcould* be realized in 12 h. 

**Figure 10:**
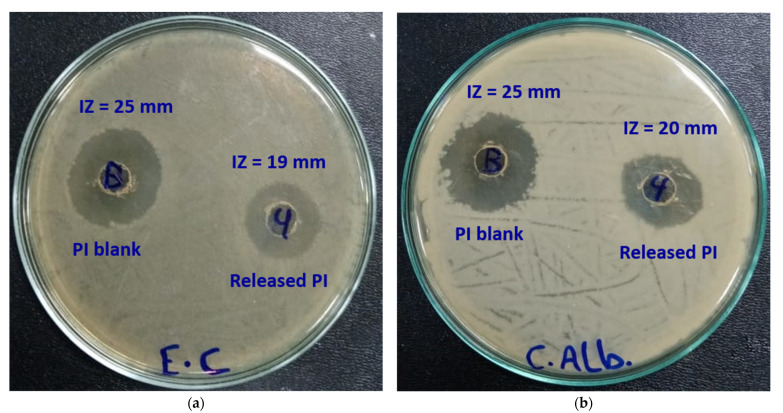
Biological activities of PI (left) and released PI (right) against; (**a**) *E. coli* and (**b**) *C. albicans*.

The antimicrobial activity of the released PI was tested to examine the effect of release on the activity of PI. *Escherichia coli* ATCC-25922 (*E. coli*, gram—ve bacteria) and *Candida albicans* ATCC-10231 (*C. albicans*, fungi) were the selected pathogens. Due to the slowest release, the PI released from Pe@CMP2 after 4 h was chosen. In addition, the pure PI (10%) was used for comparison the activity. The antimicrobial results (Figure 10) showed that the inhibition zone diameters were 25 mm for both pathogens in case of pure PI. While in case of the release PI, the diameter of inhibition zone was reduced to 19 mm for *E. coli* and 20 mm for *C. albicans.* The lowering in inhibition zone is logically related the concentration of PI which is lower in case of the released samples. This means that the activity of PI was not affected after releasing except the diminishing in PI concentration which logically led to decrement in inhibition zone. The data concluded that, the PI was still very active after releasing from the hydrogel and, hence, the slow-release character maximizes the benefit from all the loaded PI to be active for longer time.

## 4. Conclusions

The current research focused on the preparation of biodegradable networked hydrogel-based Pe@CMP for the controllable release of PI as a common surficial antiseptic reagent. CMP was firstly prepared from interaction of pullulan with monochloroacetic acid with different ratios. The Pe@CMP hydrogel was secondly synthesized by crosslinking of CMP with pectin in presence of cross-linker. The COOH content and DS for CMP were 24.2–51.2 mmol/kg and 0.44–0.93, respectively. By increment the pectin ratio, the rheological properties of the Pe@CMP hydrogel increased. Due to the formation of hydrogen bonding with the Pe@CMP hydrogel, the swelling ratio in water (16.0–18.0%) was higher than that of artificial sweat (11.7–13.2%). The Pe@CMP2 hydrogel showed the slowest release of PI and 57.7% of PI was released within 360 min. Kinetically, the release of PI from hydrogel was fitted to first order which explained that the release was depended on PI concentration in the hydrogel. The release was explained by the swelling mechanism as when the hydrogel swollen, the networked structures opened and, consequently, the PI release. The PI was released from the outer pores followed by inner pores and then from the bulk pores, achieving the control release behavior. The biological activity of PI showed that the released PI still very active. In addition, its biocompatibility and biodegradability, the synthesized Pe@CMP hydrogel exhibited controllable release of PI antiseptic and regulate its benefits in the treatment of the skin. The control release behavior of the prepared hydrogel could be applied as drug delivery for other skin surficial treatment. Moreover, the synthesized hydrogels could be potentially applied in bandage like sandwich between two layers from nonwoven and cotton gauze. 

## Figures and Tables

**Table 1:** Content of carboxylic group in pullulan after etherification.

Sample	Pullulan:CAA Ratio (wt:wt)	Carboxyl Content (mmol/kg)	Degree of Substitution
**Pullulan**	1.0:0.0	2.2 ± 0.6	-
**CMP1**	1.0:1.0	24.2 ± 1.8	0.42
**CMP2**	1.0:1.5	38.5 ± 2.2	0.66
**CMP3**	1.0:2.0	51.2 ± 2.7	0.88

## Data Availability

The data are available from the corresponding author upon request.

## Supplementary Materials

The following are available online at https://www.mdpi.com/article/10.3390/polym13183118/s1, Figure S1: EDX for the prepared hydrogel; Figure S2: (a) Solution uptake in NaCl and (b) swelling ratio in NaCl. Figure S3: UV spectra for the PI; (a) pure PI (different concentrations) and (b)released PI from Pe@CMP hydrogel at different time. Table S1: Parameters of kinetics for the release of betadine from the Pe@CMP composites.
