# Supplementary material for: Controllable Release of Povidone-Iodine from Networked Pectin@Carboxymethyl Pullulan Hydrogel
Source: Polymers (Basel). 2021 Sep 15;13(18):3118. doi: 10.3390/polym13183118 (PMC8468881; doi:10.3390/polym13183118)
Supplement: Supplementary file 1 [file polymers-13-03118-s001.zip › polymers-1358901-supplementary.pdf]

Supplementary File

Figure S1: EDX for the prepared hydrogel; **[a]** CMP, **[b]** Pe@CMP (1:9), **[c]** Pe@CMP (2:8), **[d]** Pe@CMP (3:7) and **[e]** PI@Pe@CMP (3:7).

[a]

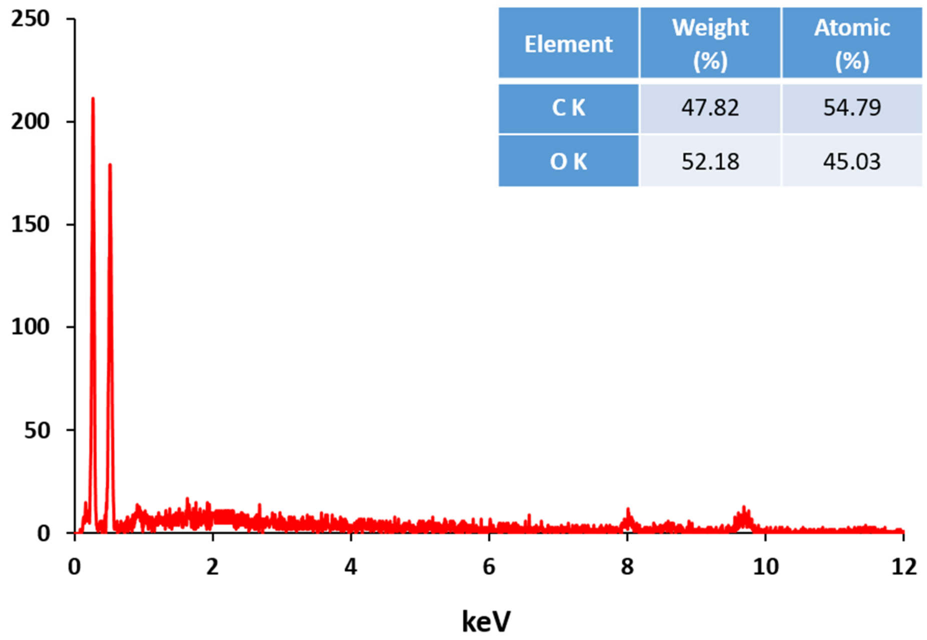

Element	Weight (%)	Atomic (%)
C K	47.82	54.79
O K	52.18	45.03

[b]

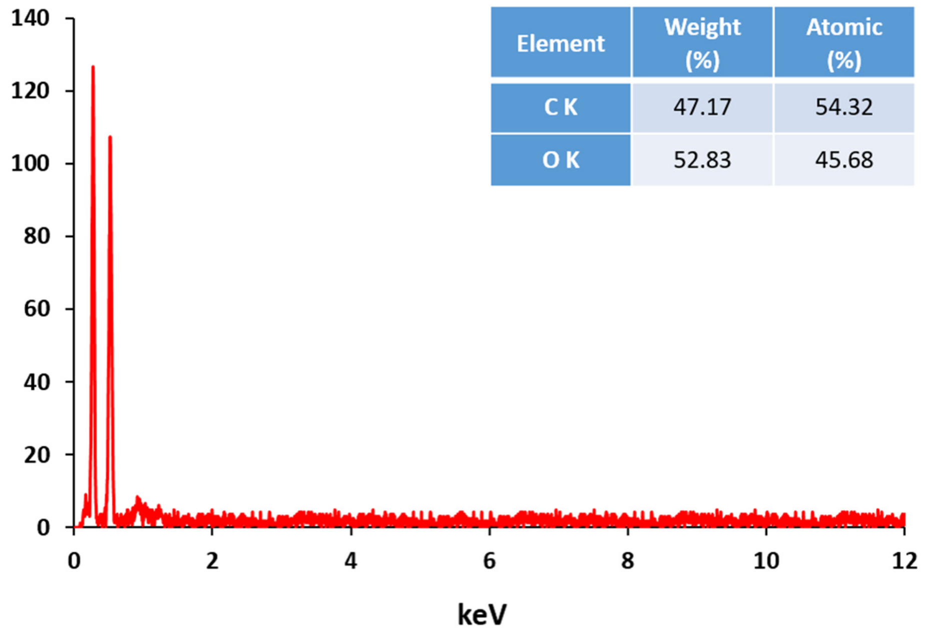

Element	Weight (%)	Atomic (%)
C K	47.17	54.32
O K	52.83	45.68

[c]

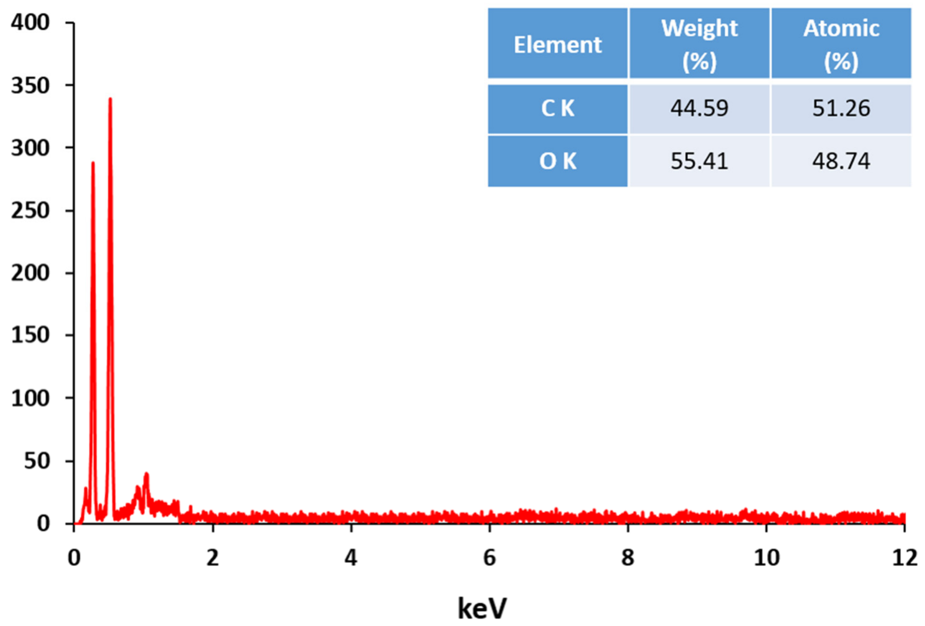

Element	Weight (%)	Atomic (%)
C K	44.59	51.26
O K	55.41	48.74

[d]

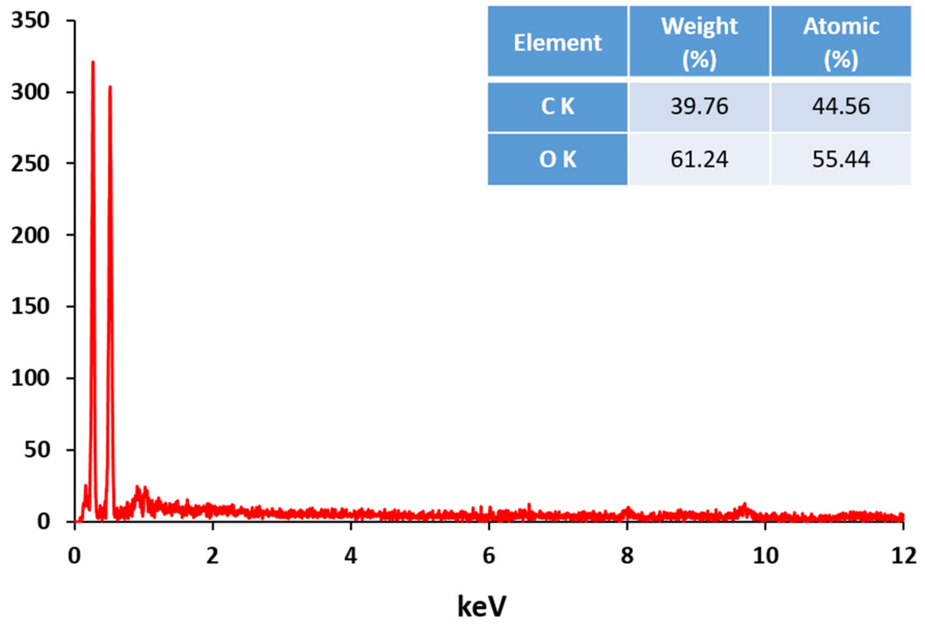

Element	Weight (%)	Atomic (%)
C K	39.76	44.56
O K	61.24	55.44

[e]

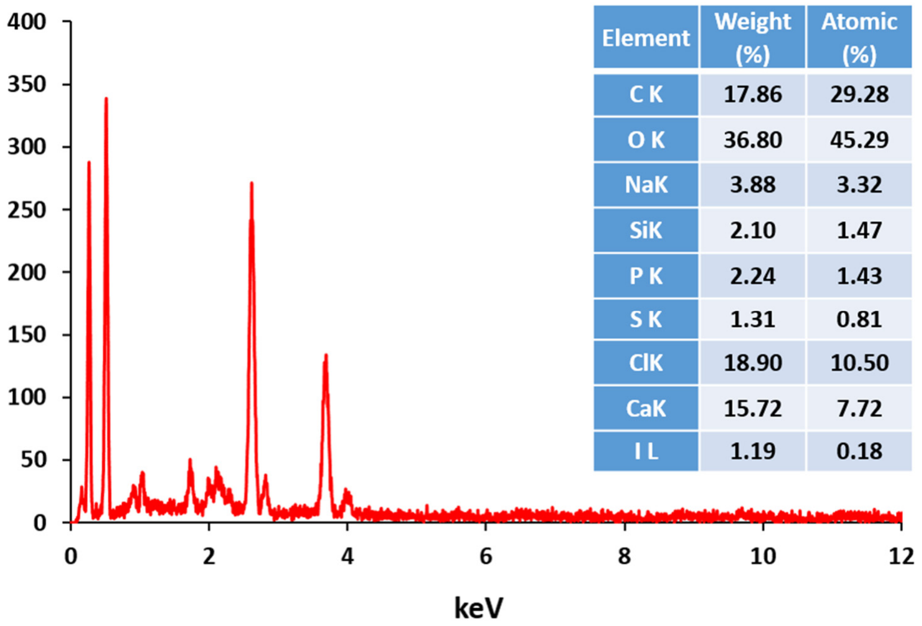

Element	Weight (%)	Atomic (%)
C K	17.86	29.28
O K	36.80	45.29
NaK	3.88	3.32
SiK	2.10	1.47
P K	2.24	1.43
S K	1.31	0.81
ClK	18.90	10.50
CaK	15.72	7.72
I L	1.19	0.18

Figure S2: [a] Solution uptake in NaCl and **[b]** swelling ratio in NaCl.

[a]

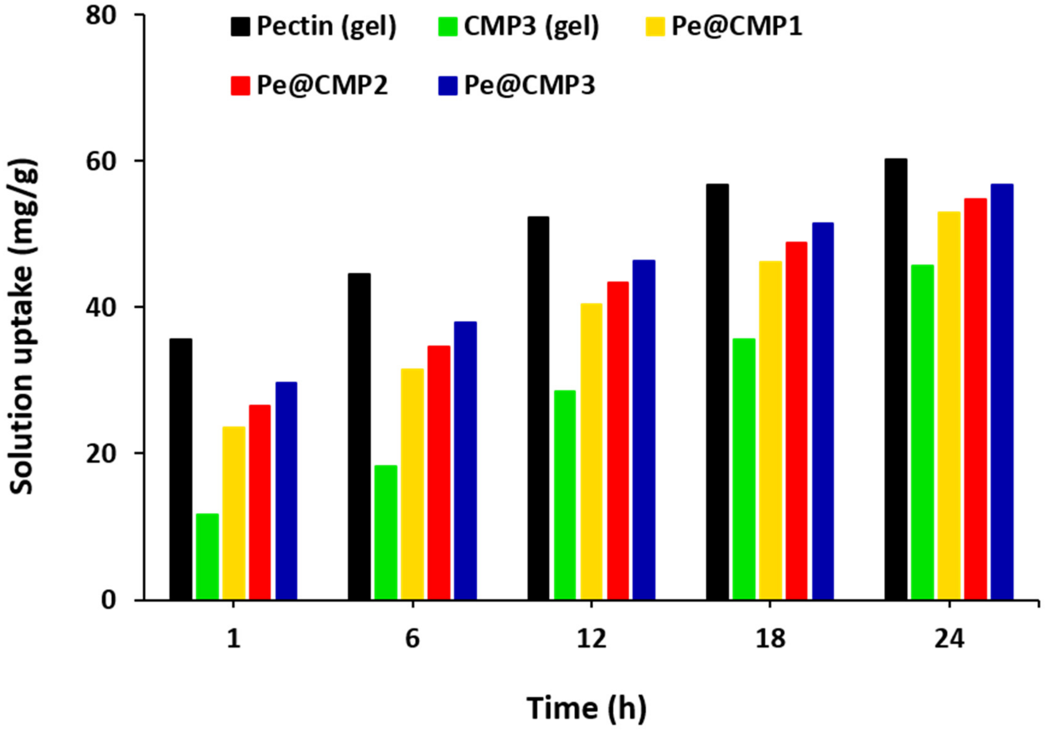

[b]

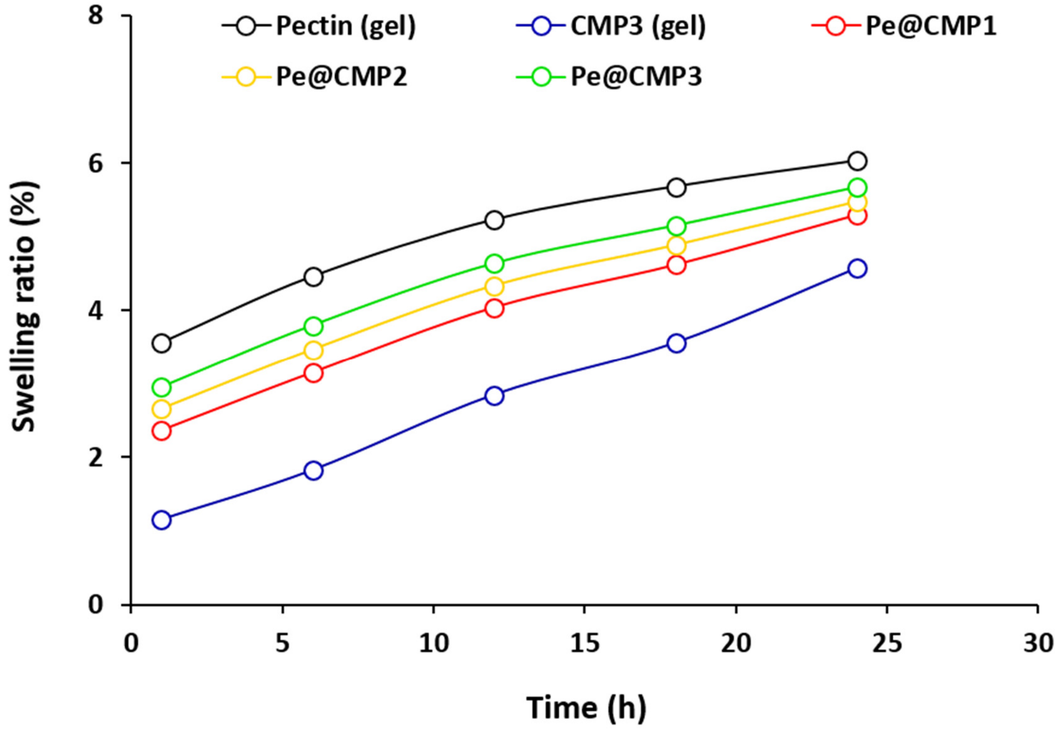

Figure S3: UV spectra for the PI; **[a]** pure PI (different concentrations) and **[b]** released PI from Pe@CMP hydrogel at different time.

[a]

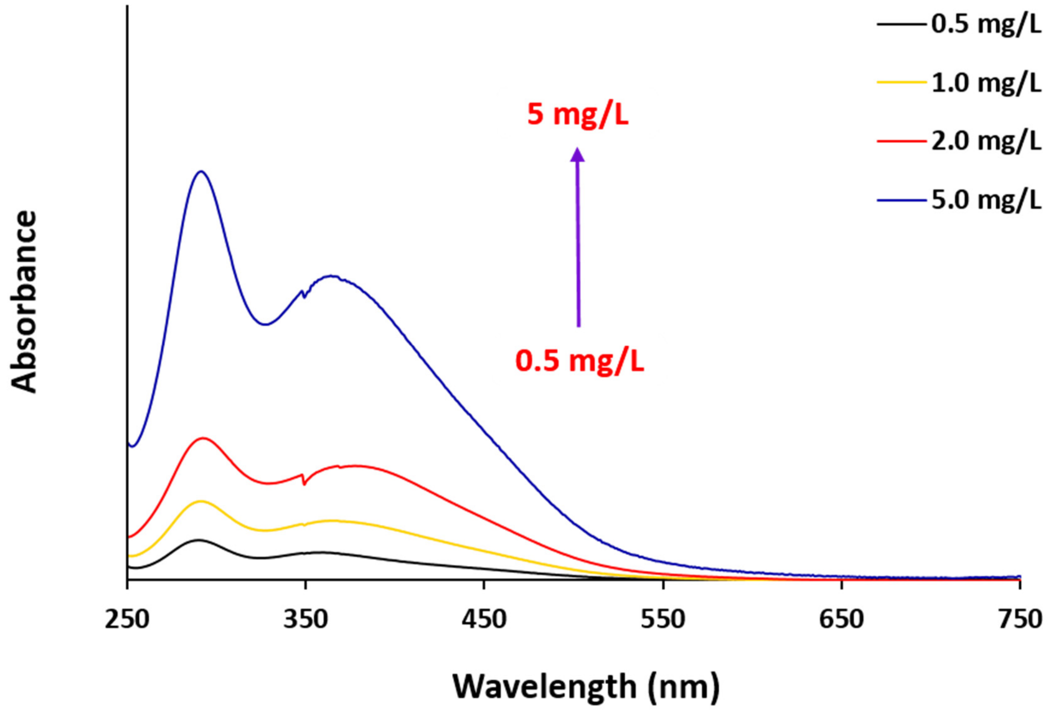

[b]

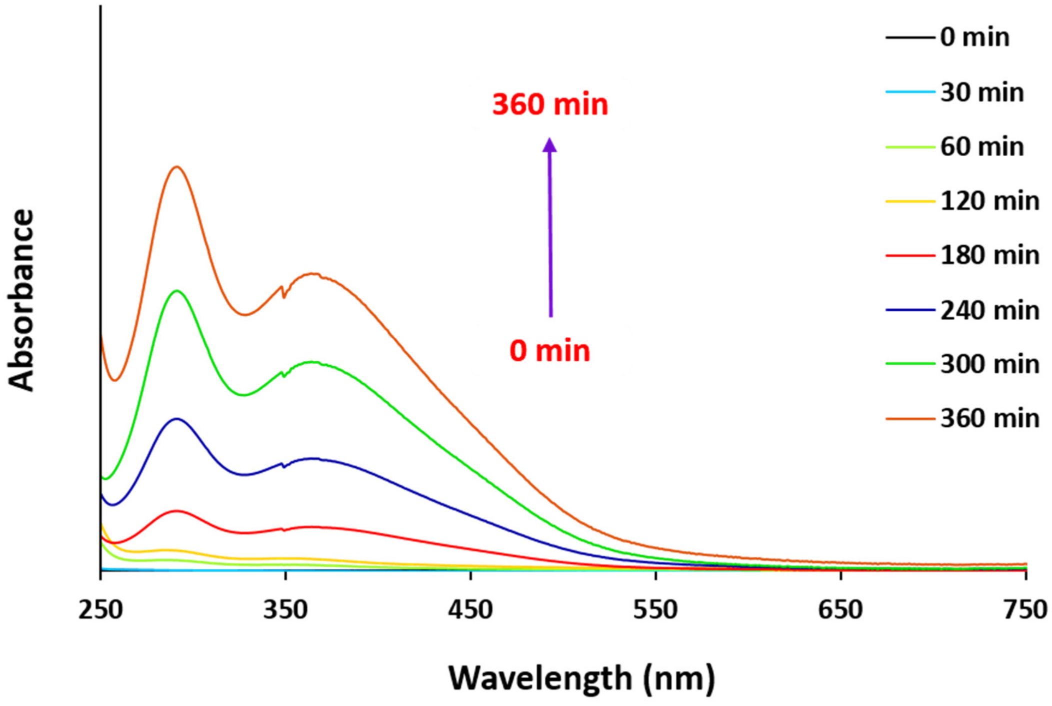

Table S1: Parameters of kinetics for the release of betadine from the Pe@CMP composites.

Samples	First Order		Higuchi		Hixson-Crowell		Korsmeyer-Peppas		
	R^2	K_1 (h^{-1})	R^2	K_H ($h^{-1/2}$)	R^2	K_{HC} x 10^{-3} ($h^{-1/3}$)	R^2	n	K_{KP} x 10^{-3} (h^{-n})
**CMP**	0.98	2.75	0.94	14.49	0.96	192.0	0.98	0.069	300.0
**Pe@CMP1**	0.95	1.69	0.90	9.19	0.87	78.0	0.94	0.051	186.0
**Pe@CMP2**	0.99	0.79	0.92	5.96	0.95	48.0	0.99	0.049	84.0
**Pe@CMP3**	0.90	1.37	0.88	7.62	0.75	54.0	0.87	0.077	150.0
